# Response of the Intertidal Microbial Community Structure and Metabolic Profiles to Zinc Oxide Nanoparticle Exposure

**DOI:** 10.3390/ijerph17072253

**Published:** 2020-03-27

**Authors:** Yinghai Wu, Xinyu Rong, Cuiya Zhang, Renduo Zhang, Tao He, Yunjun Yu, Zhuangming Zhao, Jing Yang, Rui Han

**Affiliations:** 1 Dalian Ocean University, Dalian 116023, China; wuyinghai@dlou.edu.cn (Y.W.); r0807xy@163.com (X.R.); zhangcuiya@dlou.edu.cn (C.Z.); 2 South China Institute of Environmental Science, Ministry of Ecology and Environment, Guangzhou 510655, China; hetao@scies.org (T.H.); yuyunjun@scies.org (Y.Y.); zhaozhuangming@scies.org (Z.Z.); 3 Guangdong Provincial Key Laboratory of Environmental Pollution Control and Remediation Technology, Sun Yat-sen University, Guangzhou 510275, China; zhangrd@mail.sysu.edu.cn; 4 Key Laboratory of Environment Controlled Aquaculture, Ministry of Education, Dalian 116023, China

**Keywords:** intertidal zone, ZnO NPs, microbial distribution, carbon metabolism, sediment

## Abstract

The toxicity of nanomaterials to microorganisms is related to their dose and environmental factors. The aim of this study was to investigate the shifts in the microbial community structure and metabolic profiles and to evaluate the environmental factors in a laboratory scale intertidal wetland system exposed to zinc oxide nanoparticles (ZnO NPs). Microbial assemblages were determined using 16S rRNA high-throughput sequencing. Community-level physiological profiles were determined using Biolog-ECO technology. Results showed Proteobacteria was the predominant (42.6%–55.8%) phylum across all the sediments, followed by Bacteroidetes (18.9%–29.0%). The genera *Azoarcus*, *Maribacter*, and *Thauera* were most frequently detected. At the studied concentrations (40 mg·L^−1^, 80 mg·L^−1^, 120 mg·L^−1^), ZnO NPs had obvious impacts on the activity of *Proteobacteria*. Adverse effects were particularly evident in sulfur and nitrogen cycling bacteria such as *Sulfitobacter*, unidentified*_Nitrospiraceae*, *Thauera,* and *Azoarcus*. The alpha diversity index of microbial community did not reflect stronger biological toxicity in the groups with high NP concentrations (80 mg·L^−1^, 120 mg·L^−1^) than the group with low NP concentration (40 mg·L^−1^). The average well color development (AWCD) values of periodically submersed groups were higher than those of long-term submersed groups. The group with NP concentration (40 mg·L^−1^) had the lowest AWCD value; those of the groups with high NP concentrations (80 mg·L^−1^, 120 mg·L^−1^) were slightly lower than that of the control group. The beta diversity showed that tidal activity shaped the similar microbial community among the periodically submerged groups, as well as the long-term submerged groups. The groups with high DO concentrations had higher diversity of the microbial community, better metabolic ability, and stronger resistance to ZnO NPs than the groups with a low DO concentration.

## 1. Introduction

Metal oxide nanoparticles (MONPs) can be broadly defined as metal oxide particles with at least one dimension between 1 and 100 nm [1]. Owing to their distinctive physical, chemical, and biological properties, MONPs have increasing industrial applications. In particular, zinc oxide nanoparticles (ZnO NPs) are widely used in products such as electronic sensors, solar voltaics, transducers, plastics, ceramics, glass, cement, rubber, lubricants, paints, pigments, photocatalysts, personal care products, etc. [2,3]. The increasing application of ZnO NPs has increased their environmental release, raising concerns about their potential ecological damage [4,5].

Current estimates of ZnO NP concentrations in natural surface water and treated wastewater range from less than 100 ug/L to 430 ng/L and reach a few mg/kg in sludge-treated soil [6,7]. Environmental levels of ZnO NPs are expected to increase continually given the accumulation from various discharge sources. ZnO NPs in the environment involve both soluble and particulate species because of partial solubility, suggesting that toxic action can be given full play [5]. Coastal wetlands could be a major sink of ZnO NPs released to the environment owing to the transport downstream and the potential 250 tons of sunscreen washed off each year, which is the intersection of terrestrial and marine pollution [8,9,10]. The microbial resources in coastal wetlands are of great significance for intertidal aquaculture, ecological balance, and pollutant degradation in the ocean and are among the most abundant and diverse groups on earth [11]. To understand the ecological damage caused by ZnO NPs, their effects on the microbial communities in coastal wetlands must be addressed, and the changes in carbon metabolism should also be investigated. However, information on ZnO NP effects on the microbial communities and carbon metabolism in coastal wetlands is limited [4,5].

Most NP toxicity studies on ecologically relevant bacterial species have been conducted on *E. coli*, *Bacillus subtilis*, *Pseudomonas putida*, *Shewanella oneidensis*, etc. [5]. Growth inhibition and cell viability are usually used as endpoints. Some studies have examined the effect of particle size on the antibacterial activity of ZnO and reported different findings [12,13,14]. The concentration of ZnO NPs also had an important effect on microbes; the presence of 10 and 50 mg/L of ZnO NPs decreased total nitrogen removal efficiencies, from 81.5% to 75.6% and to 70.8%, respectively, in a sequencing batch reactor [15]. Several studies on bacteria that attempted to understand the impact of water chemistry on the bioavailability and toxicity of ZnO NPs found that Zn^2+^ dissolution, pH, the amounts of reactive oxygen species (ROS), and organic matter were the key factors affecting the toxic effect of ZnO NPs on cultured bacteria [16,17,18]. The water in the intertidal zone fluctuates periodically, so the sediments are periodically submerged or exposed to the air. The physical–chemical indexes such as dissolved oxygen (DO), total organic carbon (TOC) and nitrogen may also affect the ecotoxicity of nanomaterials. The sparse literature has investigated effects of ZnO NPs under environmentally-relevant settings involving bacterial communities; these particles reduced microbial biomass and diversity and significantly changed soil enzymes [6,19]. It is difficult to extrapolate the conclusions obtained from pure culture studies to complex microbial communities and the functions of microbial communities in coastal wetlands affected by tides.

The objectives of the present study were as follows: i) to investigate the effect of different concentrations of ZnO NPs on the intertidal microbial community structure and community-level physiological profiles (CLPP) and to evaluate the impact of key environmental factors on the ecotoxicity of ZnO NPs.

## 2. Materials and Methods

### 2.1. Reactor, Operation, and Sampling

**Figure 1:**
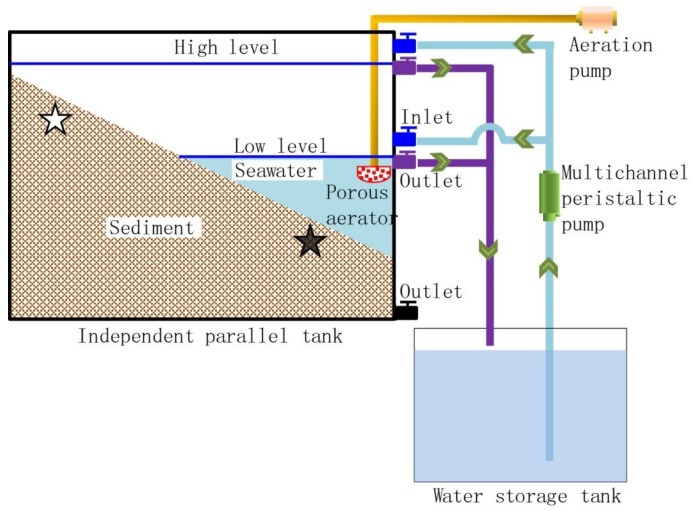
The lab-scale simulated intertidal system, operation process, and sampling locations. Arrows show the seawater flow direction in the system. The white star is the location of periodically submerged samples; the solid star is the location of long-term submerged samples.

A lab-scale simulated intertidal system was established, which included four independent parallel tanks made of a polymethyl methacrylate plate, four water storage tanks, a multichannel peristaltic pump, an aeration pump, silica gel tube, inlets, outlets, sediment, and seawater (Figure 1). Each independent parallel tank had a volume of 0.03 m^3^ (1 × 0.06 × 0.5 m). The sediment and seawater were collected from an intertidal zone (121°33′34.08″ E, 38°52′9.78″ N) located Dalian city, Liaoning Province, China. Sediments were sampled from five positions at a 0.5 m horizontal interval to make the samples representative. The sediments were collected manually at a depth of 0–40 cm with an iron shovel. Seawater was collected and stored in a disinfected plastic bucket. The sediments and the seawater were transported to the laboratory immediately and were preserved in ice in the dark. The sediments from different depths were thoroughly mixed, in quadruplicate, and were used to fill four parallel flumes, similar to the natural intertidal sediment.

Commercial ZnO NPs (Alladdin, Shanghai, China) were used in the experiments, and the characterization is shown in Table S1 in Supplementary Information (SI). The concentration of the ZnO NP stock suspension was 3 g·L^−1^. A ZnO NP suspension was added after the simulated intertidal system was running for a period of 15 days; the concentration was 0, 40, 80, and 120 mg·L^−1^ in the four respective independent parallel tanks. The four independent parallel tanks were numbered C, T1, T2, and T3. The NP stock suspension was prepared by dispersing ZnO NPs in seawater, followed by 1 h of ultrasonication (25 °C, 250 W, 40 Hz) before use. The experimental system worked at a temperature of 9°C with two cycles each day. Each cycle (12 h) consisted of 6 h flowing and 6 h ebbing. In each cycle, seawater was first pumped into the independent parallel tank at the same velocity from its corresponding independent seawater storage tank, and the water level reached a high level and a low level by adjusting the upper and lower outlet valves, respectively. Therefore, half of the sediment was in the periodically submerged zone; the other half was in the long-term submerged zone. The low water level occurred at 9:30 and 21:30 daily. The water was drained out the outlet by gravity when the valve was open. When the water level dropped to the outlet level, seawater was constantly circulated in the system using a multichannel peristaltic pump. For simulating the compensation effect of waves on DO, an aeration pump was used for aeration to maintain the DO concentration (8 ± 0.2 mg·L^−1^) in seawater. Some seawater was evenly added to compensate for the evaporation from the tanks. The experiment operated for twenty-one days before sampling. 

The sediment samples were sampled from the periodically submerged zones (treatment group numbers: C.H, T1.H, T2.H, and T3.H) and long-term submerged groups (treatment group numbers: C.L, T1.L, T2.L, and T3.L) in the lab-scale simulated intertidal system; in total, 24 sediment samples were collected. Sediment samples for each group were collected at a depth of 10 cm, homogenized manually, and stored at −80 °C before use.

### 2.2. DNA Extraction and High-Throughput Sequencing

The extraction, detection, and determination of the genomic DNA of the sediment samples were performed according to a previous study [20]. The extracted genomic DNA was checked by 1.5% agarose gel electrophoresis.

Primers for sequencing were 515F (5′-GTG CCA GCM GCC GCG GTA A-3′) and 806R (5′-GGA CTA CHV GGG TWT CTA AT-3′), with different barcodes for the V4 region of the bacterial and archaeal 16S rRNA gene [21]. The details of the PCR mixture and PCR procedure can be seen in a previous study [20]. A mixture of the amplicons was then used for sequencing on the Illumina MiSeq platform (paired-end 250-bp mode) by the Beijing Novogene Technology Co., Ltd (Beijing, China).

### 2.3. High-Throughput Sequencing Data Analysis

Reads with a quality less than 30 at the 3′ end were trimmed. The quality sequences were clustered into operational taxonomic units (OTUs) at the 97% similarity level. Taxonomic assignment was determined at the 80% threshold. The details of high-throughput sequencing data analysis can be seen in [20].

The relative abundance (%) of individual taxa within each community was estimated by comparing the number of sequences assigned to a specific taxon versus the number of total sequences obtained for a sample. Calculations of alpha-diversity (the indexes used to estimate the number of OTUs in a community, including Chao1, observed species, ACE, PD_whole_tree, and Simpson) and beta-diversity (weighted Unifrac-based) metrics were done according to [20]. Weighted Unifrac-based principal coordinate analysis (PCoA) was used to show the differences among the sediment samples. Beta diversity was also evaluated with the bacterial community data to examine the differences in community patterns via a beta diversity heatmap.

### 2.4. Microbial Metabolic Activity Analysis

Biolog Ecoplates^TM^ (Biolog, Inc., Hayward, CA), used for CLPP, contained 31 environmentally applicable substrates and one control well in three replicates (96 wells). In total, 31 single carbon sources were divided into six categories; 5 g of sediment was taken from eight groups of stable systems. The sediment was placed in a 45 ml sterile saline solution (0.9% NaCl solution) for ultrasonic vibration (30 min, 250 rpm/min). After standing for 10 min, 5 ml supernatant was diluted 1000 times with sterile normal saline. The suspension was diluted with sterile saline to 0.06 at the optical density of 600 nm (OD_600_) by an ultraviolet spectrophotometer. The whole operation process was carried out under sterile conditions. The diluted suspension was added to the Biolog-ECO plates preheated at 25 °C, at 150 μL per pore, covered and incubated in a 25 °C incubator for 328 hours. The absorbance at 590 nm wavelength was determined using a microplate reader every 12 hours during incubation. Each sample was repeated three times.

### 2.5. Physicochemical Analysis

The sediment samples were used to measure the temperature, pH value, DO, salinity, ammonia nitrogen (NH_4_-N) and nitrate nitrogen (NO_3_-N). The measurements were repeated three times for each sample. Temperature, pH, salinity, and DO were measured in situ using a portable multi-parameter monitor (HACH sensION+MM150, USA). Sediment samples for NH_4_-N and NO_3_-N analysis were homogenized and frozen as soon as possible. Sediment samples for nitrogen analysis were extracted with 1 M KCl [22]. Subsequent analyses of NH_4_-N and NO_3_-N were carried out using a continuous-flow analyzer (Flow Solution IV, OI, USA) [23].

### 2.6. Calculation and Statistical Analyses

The R package (Bell Laboratories, Murray Hill, USA) was used to analyze the differences in alpha and beta diversities between groups. A T-test and the Wilcox test were used to analyze the differences between two groups. Tukey’s test and the Wilcox test of the Agricola package were used for more than two groups. One-way analysis of variance (ANOVA) was performed to compare differences in physicochemical properties and the microbial metabolic activity of the sediment. Post-hoc tests with Duncan’s statistics at *p* = 0.05 were performed to analyze the differences between groups of data [20].

Determination of average well color development (AWCD) values was conducted as follows: The capability of microorganisms to utilize different carbon sources in microbial communities was measured by average well color development (AWCD) [24]. The ability of microbial communities in each treatment group to utilize carbon sources represents the metabolic capacity of each group of microorganisms. Samples with larger variation are thought to have a higher carbon source utilization capability and tend to have higher microbial abundance [25]. The calculation for the AWCD is:

(1) $$\mathrm{AWCD}=\sum \left(C_{i}-R\right)/n$$

In Equation (1), *C_i_* is the absorbance value of each reaction well at 590 nm, *R* is the absorbance value of the control well, *n* is the number of substrates, equal to 31 in this experiment; *C_i_*–*R* < 0.06 of wells was calculated as zero [26].

## 3. Results

### 3.1. Microbial Community Composition

In total, 1,274,783 high-quality sequences were acquired from the 24 sediment samples, with a range of 40,283 to 66,323 sequences per community (Table S2 in SI). In total, 68,983 OTUs were identified. Rarefaction analysis suggested the full extent of microbial diversity was generally high in all sediment samples (Figure S1 in SI).

**Figure 2:**
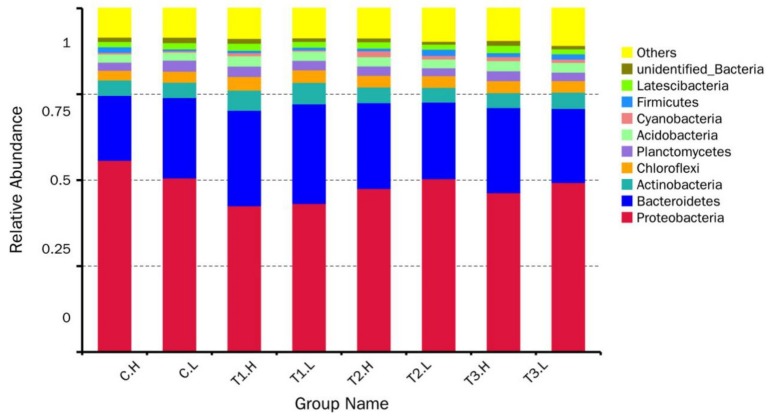
Relative abundance (%) of dominant microbial taxa across all analyzed sediments revealed by 16S rRNA MiSeq sequencing, at the phylum level, of the top 10 relative abundance values. In the group name description, the letter C indicates CK (the control); H and L indicate periodically submerged and submerged, respectively; T1, T2, and T3 indicate the three concentrations of ZnO NP, 40 mg·L^−1^, 80 mg·L^−1^, 120 mg·L^−1^. Three duplicates in each group were sampled three times from one system for each treatment and were numbered 1, 2, and 3.

In all sediment communities, *Bacteria* dominated (> 99.0%) (Figure 2). Among the nine bacterial phyla identified, *Proteobacteria* were predominant (42.6%–55.8%) across all the sediments, followed by *Bacteroidetes* (18.9%–29.0%), with other abundant phyla, such as *Actinobacteria* (4.2%–6.4%), *Chloroflexi* (2.9%–3.9%), and *Planctomycetes* (2.3%–3.2%). Remarkably, the relative abundance of *Proteobacteria* was highest (> 50%) in the control groups (C.H and C.L) and lowest in the T1 group, but with no significant difference between the T2 and T3 groups (*p* > 0.05). *Bacteroidetes* and *Acidobacteria* had the highest abundance in the T1 group. *Chloroflexi*, *Planctomycetes,* and *Acidobacteria* fluctuated among all samples. *Firmicutes* had the highest abundance in the T1, T3, and T4 groups. The abundance of *Proteobacteria* was higher in the C.H group than in the C.L group and was lower in other periodically submerged groups (T1.H, T2.H, T3.H) than in submerged ones (T1.L, T2.L, T3.L). In the control groups (C.H and C.L) and T1 groups, *Bacteroidetes* had higher abundance in the submerged group than in the periodically submerged one.

**Figure 3:**
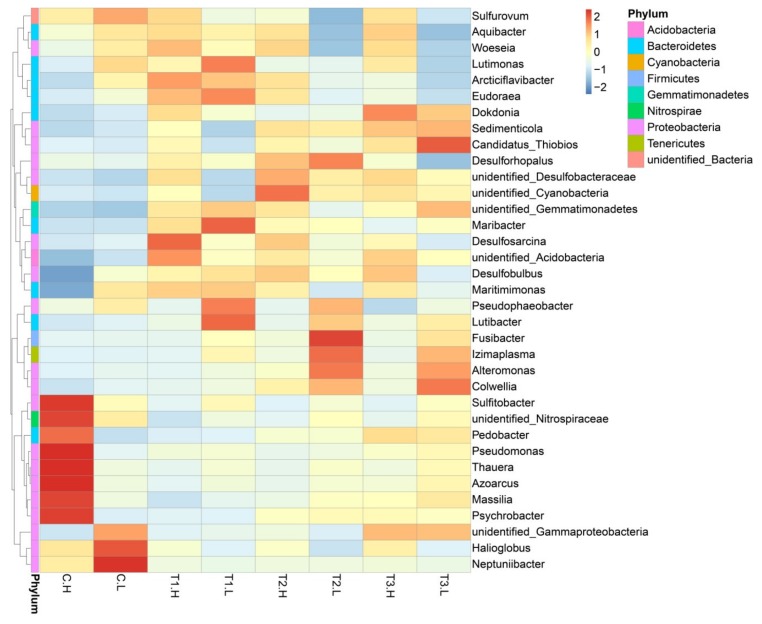
Relative abundance (%) of the top 30 microbial genera across eight groups revealed by 16S rRNA MiSeq sequencing. The sample numbers are shown in Figure 2. The unidentified genera were reconfirmed according to the species level.

At the genus level, members from *Azoarcus*, *Maribacter*, *Thauera*, *Maritimimonas*, *Woeseia*, *Desulfosarcina*, *Arcticiflavibacter*, *Massilia*, and *Aquibacter* were most frequently detected (Figure 3). *Sulfitobacter*, *unidentified_Nitrospiraceae*, *Pedobacter*, *Pseudomonas*, *Thauera*, *Azoarcus*, *Massilia*, and *Psychrobacter* showed higher abundance in the C.H group than in other groups. *Unidentified_Gammaproteobacteria*, *Halioglobus*, and *Neptuniibacter* had higher relative abundance in the C.L group than in other groups. The relative abundance of *sulfitobacter*, *unidentified_Nitrospiracae*, *Pedobacter*, *Pseudomonas*, *Thauera*, *Azoarcus*, *massilia*, *Psychrobacter*, *unidentified_Gammaproteobacteria*, *Halioglobus*, *Neptuniibacter*, *Sulfurovum*, and *Aquibater* decreased in the four treatments.

### 3.2. Microbial Community Diversity

**Figure 4:**
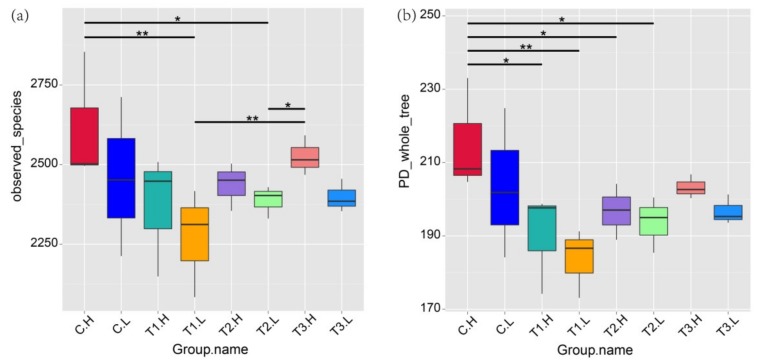
Microbial alpha diversity indexes of sediment samples. Observed_species (**a**) and PD_whole_tree (**b**). The samples numbers are shown in Figure 2. A single asterisk indicates *p* < 0.05, a double asterisk indicates *p* < 0.01. The line under the asterisk indicates that the samples below it are significantly different.

The richness and diversity indices of alpha, chao 1, ACE, and Simpson ranged from 2443 to 5648, from 2524 to 3904, and from 0.99 to 0.995, respectively (Table S2 in SI). The index of Observed_species was significantly higher in the periodically submerged group (C.H) than in the submerged group (C.L) (*p* < 0.05), as well as the TI, T2, and T3 groups (Figure 4a). The T1 group had significantly lower Observed_species values than those of the control. The T2 and T3 groups showed slight fluctuations in the Observed_species values, which also were lower than those of the control. The index of PD_whole_tree was similar to that of Observed_species; the groups with ZnO NPs had lower alpha indices (Figure 4b).

**Figure 5:**
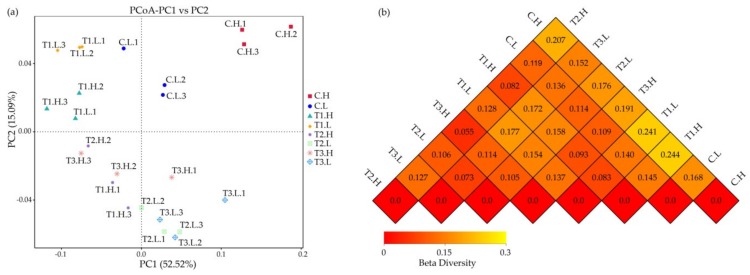
Microbial beta diversities of sediment samples. Weighted Unifrac-based PcoA (**a**) and beta diversity heatmap (**b**). The calculation of the beta diversity heatmap was based on the results of three duplicates in each group. The samples numbers are shown in Figure 2.

Results of the weighted Unifrac-based PcoA showed that the samples distributed in different parts of the data space (Figure 5a), indicating significant differences in sediment community compositions among the sampling sites. In the beta diversity analysis, the weighted Unifrac distance index was used to measure the coefficient of the difference between two samples (Figure 5b). The smaller the value, the smaller the difference between the two samples in terms of species diversity. The microbial community of the C.H group showed the most difference with that of T1.H, followed by T1.L, T2.H, T3.H, T2.L, C.L, and T3.L in turn. The microbial community of the C.L group showed the most difference with that of T1.H, followed by T1.L, T3.L, T2.H, T2.L, and T3.H in turn.

### 3.3. Microbial Metabolic Activity

**Figure 6:**
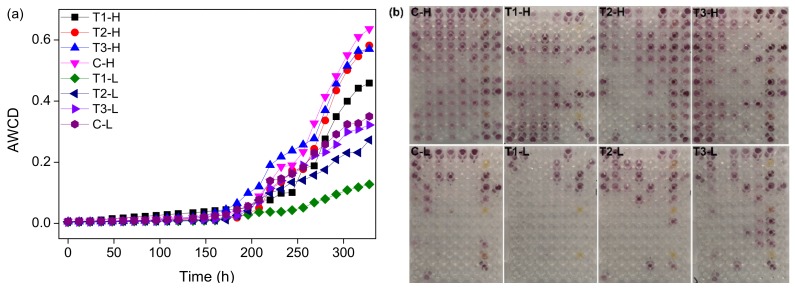
Average well color development (AWCD) of microbial communities cultured in different treatment groups (**a**) and the colour-reactions of Biolog-ECO plates in different treatment groups (**b**). The samples numbers are shown in Figure 2.

To quantify the microbial metabolic activities in the system, the AWCD inside the different treatment groups was investigated and Biolog-ECO plates appeared the colour-reactions (Figure 6). Within 196 h, the AWCD values of each treatment group treated with ZnO NPs showed little difference (Figure 6a). After 196 h, the AWCD value of all treatment groups began to rise rapidly, and after 300 h, the AWCD value increased slowly (Figure 6a). The AWCD values of periodically submersed groups were higher than those of long-term submersed groups. The T1 group had the lowest AWCD value, those of T2 and T3 were slightly lower than that of the control group.

**Figure 7:**
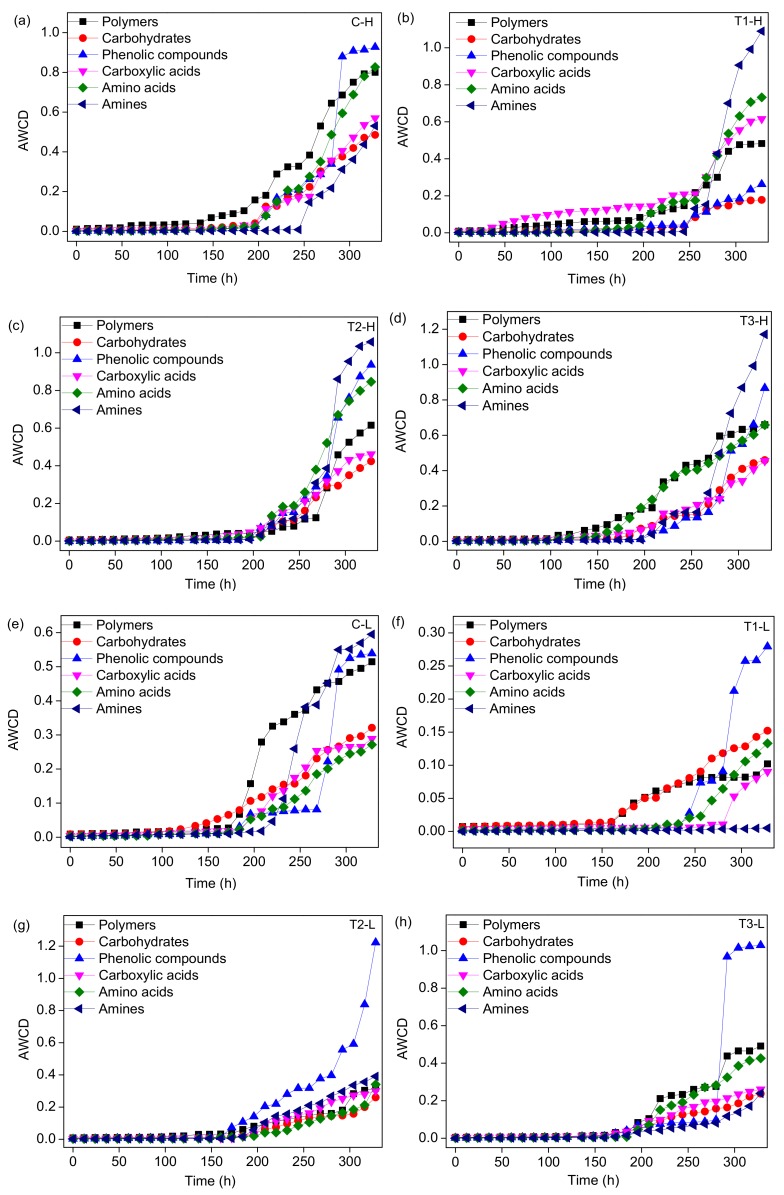
Metabolic response of micro-organisms to six carbon sources in different treatment groups. C-H group (**a**), T1-H group (**b**), T2-H group (**c**), T3-H group (**d**), C-L group (**e**), T1-L group (**f**), T2-L group (**g**), and T3-L group (**h**). The samples numbers are shown in Figure 2.

In this study, according to their biochemical properties, 31 substrates in the Biolog-ECO plates were assigned to six categories: carbohydrates, carboxylic acids, amino acids, amines, phenolic compounds, and polymers (Table S3 in SI). The average absorbance of six kinds of carbon sources varied with incubation time in the different treatment groups (Figure. 7). The carbon utilization level of the control group was different from that of the ZnO NP treatment groups. For the periodically submerged groups (Figure 7a–d), the AWCD of phenolic acids was the highest in the control group, followed by polymers and amino acids, and the AWCD of amines was the lowest (Figure 7a); a higher utilization rate of amines by microbes occurred in the treatment groups with ZnO NPS compared to the control group, while the AWCD of amino acids, polymers, and phenolic acids showed opposite trends (Figure 7a–d). For the long-term submerged groups (Figure 7e–h), the utilization of phenolic acids, amines, and polymers was higher in the control group, while that of amino acids was the lowest (Figure 7e). A higher AWCD of phenolic acids occurred in the treatment groups with ZnO NPS compared to the control group, while the AWCD of amino acids and polymers showed opposite trends (Figure 7e–h).

### 3.4. Physicochemical Properties of the Sediment

Partial physicochemical properties of the sediment are shown in Table 1. The concentration of DO in submerged groups was significantly higher than that in periodically submerged groups (*p* < 0.05). A higher concentration of NH_4_-N was found in periodically submerged groups than in submerged groups; the difference with submerged groups showed no significance. Other indicators showed no significant difference.

## 4. Discussion

### 4.1. Microbial Community Shift with Concentrations of ZnO NPs

Proteobacteria, frequently the dominant phylum in all kinds of wetlands, includes the genera *Zoogloea*, *Nitrosospira*, *Denitratisoma*, *Azonexus,* and *Azospira*, and is usually considered to play a vital role in the removal of organic matter and nitrogen [27]. Higher microbial abundances of *Proteobacteria* in the control groups than in others suggest ZnO NPs had an obvious impact on the activity of this phylum (Figure 2). The phylum Bacteroidetes is widely distributed in the environment, including in soil, sediments, and sea water, but the relative abundance of this phylum did not change in response to the ZnO NPs. The relative abundance of other phyla fluctuated in all groups and showed no regular pattern. Marine sediments constitute one of the largest biospheres in the world [28]. Studies have demonstrated that bacteriophage MS2 is less sensitive than *E. coli* to stressors such as ultraviolet (UV) exposure [29]. Therefore, the microbes in marine sediment show different characteristics and resistances to ZnO NPs, which is surprising but plausible. 

Zinc is known as an essential element for many microbes and can promote multimerization and can enhance enzymatic activity. The inhibitory effect of excessive metallic NPs on host bacterial growth could result in changes in the cell membrane property [30]. It can be seen from the genus level that more community shifts were caused by ZnO NPs than at the phylum level (Figure 3). This adverse effect was particularly evident in the sulfur and nitrogen cycling bacteria such as *Sulfitobacter*, *unidentified_Nitrospiraceae*, *Thauera,* and *Azoarcus*, which were found to play important roles in the circulation of elements in wetlands [31]. In three treatments (T1, T2, and T3) with ZnO NPs, other bacteria also showed different degrees of influence (Figure 3). A study advised to pay more attention to the effect of water chemistry on the physicochemical properties of nanoparticles in future nanotoxicity evaluations [32]. Four views were summarized on the toxicity mechanism of ZnO NPs: The reactive oxygen species (ROS) generated by nanoparticles, the release of Zn^2+^, the direct contact between nanoparticles and cells, and the internalization of ZnO NPs in cells [33]. When nanoparticles are exposed to hydroponic culture, they lose partial stability immediately and continue to form aggregates induced by divalent ions, i.e., Ca^2+^ and Mg^2+^; commercial ZnO nanoparticles aggregate slowly to a final aggregate size of about 1000 nm after 30 min [16]. In seawater, high salinity could reduce the negative mobility of particles and promote aggregation [34,35]. In saltwater, for example, the solubility of ZnO NPs was reported to be more than twice that of micron-size ZnO [10], and there is evidence that ZnO NPs will continue to dissolve until the concentration of Zn^2+^ in media reaches 3.2–4.8 mg·L^−1^ [10,35,36,37]. One or more mechanisms may be responsible for the ecotoxicity, and the dominant mechanism may depend on the test microbes and the test media. A previous study found that ZnO NPs showed a twofold increase in revertant colonies compared to the negative control in TA98, TA1537, and *E. coli* (WP2 uvrA) strains with S9 while other strains, TA100 and TA1535, did not show a significant change [38]. At half inhibitory concentration values (IC_50_) in the range of less than 1 mg·L^−1^ to several hundred mg·L^−1^ or higher, high variation exists, even in the same *E. coli* species [5]. The IC_50_ value of *Bacillus subtilis* in fresh water was lower than 1 mg·L^−1^. In contrast, the IC_50_ value of *E.coli* was almost two orders of magnitude higher [16]. In bacterial growth medium, the minimum inhibitory concentration of ZnO NPs with similar particle size (10–30 nm) to wild-type *E.coli* was as high as 500 mg·L^−1^ [39]. This means that factors such as the structure of the microbial cell membrane, concentration, and size of nanomaterials, as well as water chemistry, have important effects on the ecotoxicity of NPs. High concentrations of nanomaterials tend to lead to agglomeration of nanomaterials [2]. With the increase in nanomaterial concentrations, the alpha diversity index of the microbial community in the T2 and T3 groups did not reflect stronger biological toxicity than that in the T1 group (Figure 4a). Once in the environment, free NPs tend to form aggregates that can be trapped or eliminated through sedimentation [3]. A previous study found uptake of NPs would be necessary to ensure the availability of bare nanoparticles in the nano range without agglomeration [38]. Based on this result, ZnO NP agglomeration with high concentrations (80 mg·L^−1^, 120 mg·L^−1^) of ZnO NPs attenuated the ecotoxicity and showed similar effects to low concentrations (40 mg·L^−1^).

### 4.2. Shift of Microbial CLPP with Concentrations of ZnO NPs

In this study, 31 substrates in the Biolog-ECO plate were assigned to six categories: carbohydrates, carboxylic acids, amino acids, amines, phenolic compounds, and polymers [40]. The varying AWCD value and the final value reflected the utilization ability of microorganisms with respect to a certain carbon source. The AWCD value of each treatment group increased with the prolongation of the culture time, indicating that the microbial activity and carbon utilization capacity of each treatment group increased with the prolongation of the culture time (Figure 6). Each treatment group experienced three growth stages. At the early stage of culture, the utilization rate of the carbon source was low, and the microorganism was still in the adaptive stage (0 h–196 h); Carbon sources began to be largely utilized, and microbial growth entered the rapid growth stage (196 h–300 h); The growth of microorganisms tended to be steady, and there was no obvious decay (>300 h) in the test time (Figure 6). A previous study investigated the microbial function of soil samples using the Biolog method and found that the AWCD variation was similar to this result [41]. The addition of ZnO NPs resulted in a difference in the carbon source metabolism level, which indicated the addition of ZnO NPs weakened the microbial metabolic activity of the system. AWCD values in groups with different NP concentrations indicated the different metabolic characteristics of carbon sources [8]. This result is consistent with the shift in the microbial community obtained by high-throughput sequencing, which further suggested that ZnO NP agglomeration with high concentrations (80 mg·L^−1^, 120 mg·L^−1^) of ZnO NPs attenuated the ecotoxicity, showing similar metabolic ability to low concentrations (40 mg·L^−1^).

For different microbial communities, the capability utilizations related to the six types of carbon sources were different (Figure 7). The utilization rates of phenolic acids, amines, amino acids, and polymers were higher than the others, indicating that the microorganisms in each treatment group prefered to these four types of carbon sources in the process of growth and reproduction (Figure 7). The types of nutrients in the reaction system will stress the microbial community in the system, which results in a metabolic preference for these four types of carbon sources. Among these sources, amines exist in the tissues of various marine animals, plants, and microorganisms, especially in putrefactive proteins [42]. Microorganisms maintain normal growth, proliferation, differentiation, and acid–base balance of their cells by absorbing amines in the environment [42]. Sala et al., 2005, analyzed the functional diversity of bacteria and their utilization of different carbon sources in the western Antarctic Ocean and found that amino acids were the main carbon sources used by bacteria in the sea [43]. Amino acids are important components of organic matter, suspended and deposited particles, and dissolved organic matter in marine microorganisms [44]. Among all treatment groups, microorganisms were most sensitive to phenolic acids, amines, and polymers. For the periodically submersed treatment groups, compared to the metabolic characteristics of the control group, the utilization rates of phenolic acids and amines were seriously impacted by the addition of ZnO NPs, while the utilization rate of polymers was slightly impacted (Figure 7a–d). It is speculated that the structure of the microbial community changed due to the addition of ZnO NPs, which makes the existing microbial community have a metabolic preference for amines, phenolic acids, and polymers. For the long-term submersed groups, the addition of ZnO NPs had a slight effect on the metabolic preference for phenolic acids by microorganisms, but a serious effect on the metabolic preference for amines and polymers (Figure 7e–h).

### 4.3. The Effects of Environmental Factors on the Ecotoxicity of ZnO NPs

Environmental factors can lead to different microbial communities. The tide makes the tidal flat appear periodically submerged and exposed, which changes the growth environment conditions (DO, nutrition conditions, etc.) of the microbial community and plays an important role in the microbial biogeochemical cycle process [45]. The beta diversity showed that the microbial community in periodically submerged groups was similar, while that in long-term submerged areas was similar (Figure 5b), which is attributed to the environmental factors in different locations. Redox potential and NH_4_-N are the important factors affecting the overall microbial community patterns, and total organic carbon had a relatively high impact on some denitrifiers in a terrestrial constructed wetland system [31]. In this study, the concentration of DO in periodically submerged groups was obviously higher than that in submerged groups. The concentration of NH_4_-N in periodically submerged groups was also higher than that in submerged groups, while the difference between periodically submerged groups and submerged groups was not significant (*p* < 0.05) with respect to NH_4_-N. Other indicators showed no obvious differences. It is noteworthy that the groups with high DO and NH_4_-N concentrations had higher microbial community diversity and relatively strong resistance to ZnO NPs. Therefore, DO was the most important factor shaping the microbial community in this study, followed by NH_4_-N. For the groups with high microbial community diversity, the resistance of the microbial community to environmental pressure and environmental difference was relatively strong, which is an important regulatory mechanism for the microbial community to adapt to environmental changes. ROS and Zn^2+^ associated with ZnO NPs are important factors responsible for biological toxicity. ROS has strong oxidability, which will cause damage to nucleic acids, proteins, biofilms, and other biological macromolecules and can degrade organic matter and eventually lead to cell damage [46]. The organic acid concentration and the pH were the most significant factors (*p* < 0.001) influencing aggregation and dissolution of ZnO NPs, respectively. The electrolyte type and the salt content were the next most important factors affecting both the aggregation and dissolution [47]. Water chemistry such as pH and ionic strength can significantly affect ZnO NP behavior in aqueous media [48]. Therefore, the existing form of ZnO NPs was affected by environmental factors, e.g., DO and NH_4_-N in this study, which led to different toxicities of ZnO NPs.

The metabolic function of microorganisms may also be influenced by environmental factors. The environmental and nutritional conditions of tidal flat wetlands change with the tidal phenomenon, which will affect the potential respiration of the tidal flat environment [49]. The AWCD values of the periodically submersed groups were higher than those of the long-term submersed groups. There were obvious differences in the metabolism of carbon sources among microorganisms in the long-term and periodically submersed treatment groups, indicating the submergence time impacted microbial diversity and metabolic activity. The characteristics of microbial communities of the submerged and periodically submerged groups were impacted by the addition of ZnO NPs. Long-term submergence caused the microbial function, metabolic activity, and specific utilization of the carbon sources to change [1]. The differences in temperature, pH, salinity, and NH_4_-N concentration in the periodically submersed and submersed groups were not significant (Table 1). It is speculated these four factors have little influence on the metabolism of microorganisms. The concentration of DO in the periodically submerged treatment group was significantly higher than that in the submerged group. The groups with high DO concentration had higher metabolic capacities of the 31 carbon sources than the groups with low DO concentration (Figure 6a), suggesting that the high DO concentration encouraged microbes to metabolize various carbon sources. For example, the metabolism of amines, amino acids, and polymers in the groups with high DO concentration was higher than that in the groups with low DO concentration (Figure 7). A previous study investigated the microbial communities in salt marsh segments and found that surface and deep segments presented different metallic profiles in terms of the ECO-carbo source utilization pattern [50]. This result is similar to ours. It is suggested that the groups with higher DO concentration and better metabolic ability had a stronger tolerance to ZnO NPs than the groups with low DO concentration.

## 5. Conclusions

This study investigated the effect of different concentrations of ZnO NPs on the intertidal microbial community structure and CLPP and evaluated the impact of key environmental factors on the ecotoxicity of ZnO NPs. *Proteobacteria* was the predominant (42.6%–55.8%) phylum across all the sediments, followed by *Bacteroidetes* (18.9%–29.0%). The genera *Azoarcus*, *Maribacter*, and *Thauera* were most frequently detected. The microbes in marine sediments have different characteristics and resistances to ZnO NPs. The alpha and beta diversities indicated the addition of ZnO NPs can cause significant differences in sediment community compositions among the sampling sites. Nevertheless, ZnO NP agglomeration with high concentrations (80 mg·L^−1^, 120 mg·L^−1^) of ZnO NPs attenuated the ecotoxicity. The result is consistent with the shift in the metabolic characteristics obtained by the Biolog method. Microbes were most sensitive to phenolic acids, amines, and polymers through the addition of ZnO NPs. It is speculated that the structure of the microbial community has changed due to the addition of ZnO NPs, which shifted the metabolic preference of the existing microbial community with respect to amines, phenolic acids, and polymers. Submergence time shaped the microbial community and carbon utilization capacity. DO and NH_4_-N can contribute to the high diversity of the microbial community, good metabolic ability, and strong resistance to ZnO NPs. Both the downstream transport of ZnO NPs and the widespread use of sunscreen containing ZnO NPs can reach marine microbes easily. These high exposures may be sporadic and acute but have an impact on marine organisms along the coastline. This study will help us to understand the toxicity of ZnO NPs to organisms in intertidal wetlands.

## Figures and Tables

**Table 1:** Mean values of partial physicochemical parameters of sediments in the experimental coastal wetland system (in group name, letter L: submerged; H: periodically submerged). *n* = 3.

Group Name	Temperature (°C)	DO (mg·L^−1^)	pH	Salinity (PPT)	NH_4_-N (mg·L^−1^)
C-L	11.1	0.22	8.29	5.77	0.11
T1-L	11.2	0.19	8.29	5.92	0.17
T2-L	11.1	0.20	8.28	5.96	0.11
T3-L	11.0	0.20	8.30	5.98	0.11
C-H	10.1	6.60	8.33	5.97	0.11
T1-H	10.0	6.52	8.34	5.80	0.21
T2-H	10.2	6.55	8.33	5.79	0.17
T3-H	10.1	6.56	8.34	5.83	0.08

## Supplementary Materials

The following are available online at https://www.mdpi.com/1660-4601/17/7/2253/s1, Figure S1. α-Diversity comparison; Table S1. Characterization of ZnO NPs used in the study; Table S2. Summary of 16S RNA Miseq sequences, operational taxonomic units (OTUs), and microbial diversity of sediment samples; Table S3. Carbon sources in Biolog Ecoplates.
