# Supplementary material for: Response of the Intertidal Microbial Community Structure and Metabolic Profiles to Zinc Oxide Nanoparticle Exposure
Source: Int J Environ Res Public Health. 2020 Mar 27;17(7):2253. doi: 10.3390/ijerph17072253 (PMC7177635; doi:10.3390/ijerph17072253)
Supplement: Supplementary file 1 [file ijerph-17-02253-s001.pdf]

International Journal of
*Environmental Research and Public Health*

MDPI

# Supplementary Material

*Article*

# Response of the Intertidal Microbial Community Structure and Metabolic Profiles to Zinc Oxide Nanoparticle Exposure

**Yinghai Wu [1,2,3], Xinyu Rong [1], Cuiya Zhang [1], Renduo Zhang [3], Tao He [2], Yunjun Yu [2], Zhuangming Zhao [2], Jing Yang [2,*] and Rui Han [1,4,*]**

[1] Dalian Ocean University, Dalian 116023, China; wuyinghai@dlou.edu.cn (Y.W.); r0807xy@163.com (X.R.); zhangcuiya@dlou.edu.cn (C.Z.)
[2] South China Institute of Environmental Science, Ministry of Ecology and Environment, Guangzhou 510655, China; hetao@scies.org (T.H.); yuyunjun@scies.org (Y.Y.); zhaozhuangming@scies.org (Z.Z.)
[3] Guangdong Provincial Key Laboratory of Environmental Pollution Control and Remediation Technology, Sun Yat-sen University, Guangzhou 510275, China; zhangrd@mail.sysu.edu.cn
[4] Key Laboratory of Environment Controlled Aquaculture, Ministry of Education, Dalian 116023, China
* Correspondence: yangjing@scies.org (J.Y.); hanrui@dlou.edu.cn (R.H.); Tel.: +86-20-29119533 (J.Y.); +86-411-84763257 (R.H.)

Received: 22 February 2020; Accepted: 24 March 2020; Published:

International Journal of
*Environmental Research and Public Health*

MDPI

**Table S1.** Characterization of ZnO NPs used in the study. Product No.: Z112847, CAS No.: 1314-13-2.

| Test | Specification | | Target value |
|---|---|---|---|
| | **Min** | **MAX** | |
| Appearance | white powder | | |
| Solubility | low solubility in water or ethanol; soluble in acid, ammonia, and alkali hydroxide | | |
| Shape | spheroidal | | |
| Average Particle Size | 20 nm | 40 nm | 40 nm |
| Purity (Based on Trace Metal Analysis) | 49075 | 2808 | 100% |
| Total Metallic Impurities | 55385 | 2931 | 1000 ppm |
| Zn (Complexocetric Titration) | 79.1% | 81.5% | 81.5% |

International Journal of
*Environmental Research and Public Health*

MDPI

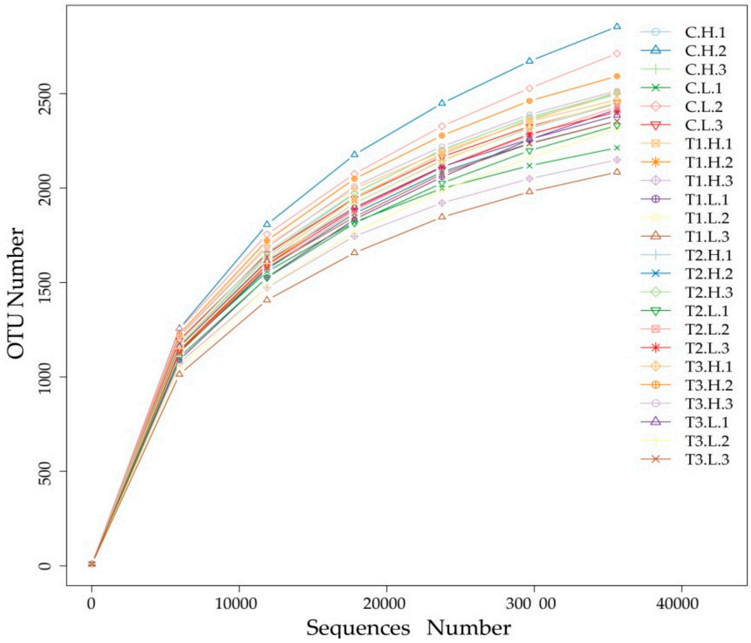


**Figure S1.** Rarefaction curves for operational taxonomic unit (OTU) comparison. The curves for OTU number were calculated using Mothur (v1.27.0) using a 0.03 distance for OTUs. In the group name description, the letter C means CK; H and L indicate intermittently submerged and submerged, respectively; T1, T2, and T3 are the three treatments of ZnO NP concentrations, 40 mg·L$^{-1}$, 80 mg·L$^{-1}$, 120 mg·L$^{-1}$. Three duplicates in each group are numbered 1, 2, and 3.

International Journal of
*Environmental Research and Public Health*

MDPI

**Table S2.** Summary of 16S RNA Miseq sequences, operational taxonomic units (OTUs), and microbial diversity of sediment samples. The samples numbers are shown in Figure S1. Microbial alpha diversity (e.g., Chao1, PD_whole_tree, and Simpson) was estimated based on 97% OTU (operational taxonomic units) clusters.

| Sample ID | No. of Sequences | No. of OTUs | Chao1 | Observed_Species | ACE | PD_Whole_Tree | Simpson |
|---|---|---|---|---|---|---|---|
| C.H.1 | 49754 | 2990 | 2943.87 | 5118 | 3075.694 | 208.269 | 0.992 |
| C.H.2 | 61973 | 3413 | 3863.134 | 4726 | 3903.579 | 233.031 | 0.99 |
| C.H.3 | 54311 | 2997 | 2886.589 | 4746 | 3119.597 | 204.739 | 0.992 |
| C.L.1 | 43538 | 2607 | 2499.968 | 4890 | 2550.179 | 184.158 | 0.993 |
| C.L.2 | 40283 | 2712 | 5648.304 | 5145 | 3903.982 | 224.832 | 0.995 |
| C.L.3 | 51614 | 2909 | 2914.188 | 5058 | 2999.113 | 201.823 | 0.994 |
| T1.H.1 | 57537 | 2948 | 3287.002 | 4604 | 3328.091 | 197.646 | 0.993 |
| T1.H.2 | 55959 | 2894 | 2851.574 | 3795 | 3067.816 | 198.726 | 0.993 |
| T1.H.3 | 49773 | 2529 | 2442.684 | 4827 | 2524.119 | 174.231 | 0.993 |
| T1.L.1 | 66323 | 2963 | 3288.002 | 4767 | 3447.206 | 191.244 | 0.992 |
| T1.L.2 | 62148 | 2834 | 3073.965 | 4771 | 3249.89 | 186.63 | 0.992 |
| T1.L.3 | 50328 | 2467 | 2465.394 | 4849 | 2563.407 | 173.119 | 0.991 |
| T2.H.1 | 55494 | 2905 | 2857.686 | 4545 | 3089.622 | 197.039 | 0.994 |
| T2.H.2 | 50922 | 2799 | 2768.633 | 4763 | 2893.564 | 188.957 | 0.993 |
| T2.H.3 | 55120 | 2986 | 2903.529 | 4613 | 3084.858 | 204.178 | 0.994 |
| T2.L.1 | 56173 | 2782 | 2978.811 | 4516 | 3049.136 | 185.414 | 0.994 |
| T2.L.2 | 57264 | 2926 | 3152.252 | 4675 | 3242.271 | 200.459 | 0.995 |
| T2.L.3 | 53422 | 2865 | 2758.373 | 4109 | 2960.304 | 195.006 | 0.995 |
| T3.H.1 | 44028 | 2843 | 2827.177 | 4069 | 2905.582 | 200.313 | 0.995 |
| T3.H.2 | 53871 | 3100 | 2965.041 | 4325 | 3171.151 | 206.781 | 0.995 |
| T3.H.3 | 49389 | 2970 | 2899.376 | 4480 | 3025.368 | 202.651 | 0.994 |
| T3.L.1 | 49075 | 2808 | 2850.673 | 4701 | 2926.071 | 195.298 | 0.994 |
| T3.L.2 | 55385 | 2931 | 2818.876 | 4019 | 3069.817 | 201.309 | 0.994 |
| T3.L.3 | 51099 | 2805 | 2739.564 | 4751 | 2922.207 | 193.599 | 0.994 |

International Journal of
*Environmental Research and Public Health*

MDPI

**Table S3.** Carbon sources in the Biolog Ecoplates

| Carbohydrates | Carboxylic Acids | Polymers | Amino Acids |
|---|---|---|---|
| α-D-lactose | α-Ketobutyric acid | α-Cyclodextrin | L-Arginine |
| β-Methyl D-glucoside | D-Galacturonic acid | Glycogen | L-Asparagine |
| D-Cellobiose | D-Glucosaminic acid | Tween 40 | L-Phenylalanine |
| D-Mannitol | D-Malic acid | Tween 80 | L-Serine |
| I-Erythritol | Itaconic acid | | L-Threonine |
| Glucose-1-phosphate | γ-Hydroxybutyric acid | Phenolic compounds | Glycyl-L-glutamic acid |
| D-Galactonic acid-γ-lactone | Pyruvic acid methyl ester | 2-Hydroxy benzoic acid | |
| D,L-α-Glycerol phosphate | | 4-Hydroxy benzoic acid | Amines |
| N-Acetyl-D-glucosamine | | | Phenyl ethylamine |
| D,L-α-Glycerol phosphate | | | Putrecine |
